# Comparative Assessment of the Effect of Synthetic and Natural Fungicides on Soil Respiration

**DOI:** 10.3390/s120303243

**Published:** 2012-03-07

**Authors:** Angelo Stefani, Joanna D’Arc Felício, Mara M. de Andréa

**Affiliations:** 1 Lab. de Ecologia de Agroquímicos, Instituto Biológico, Av. Conselheiro Rodrigues Alves 1252, São Paulo, SP CEP 04014-002, Brazil; 2 Lab. de Química e Farmacologia de Produtos Naturais, Instituto Biológico, Av. Conselheiro Rodrigues Alves 1252, São Paulo, SP CEP 04014-002, Brazil

**Keywords:** soil bioactivity, substrate-induced respiration, chlorothalonyl, *Polymnia sonchifolia*

## Abstract

As toxic pesticide residues may persist in agricultural soils and cause environmental pollution, research on natural fungicides to replace the synthetic compounds is currently increasing. The effect of the synthetic fungicide chlorothalonil and a natural potential fungicide on the soil microbial activity was evaluated here by the substrate-induced respiration by addition of glucose (SIR), as bioindicator in two soils (Eutrophic Humic Gley—GHE and Typic Eutroferric Chernosol—AVEC). The induced soil respiration parameter was followed during 28 days after soil treatment either with chlorathalonil (11 μg·g^−1^), or the methanolic fraction from *Polymnia sonchifolia* extraction (300 μg·g^−1^), and ^14^C-glucose (4.0 mg and 5.18 Bq of ^14^C-glucose g^−1^). The ^14^C-CO_2_ produced by the microbial respiration was trapped in NaOH (0.1 M) which was changed each two hours during the first 10 h, and 1, 3, 5, 7, 14 and 28 days after the treatments. The methanolic fraction of the plant extract inhibited (2.2%) and stimulated (1.8%) the respiration of GHE and AVEC, respectively, but the synthetic chlorothalonil caused 16.4% and 2.6% inhibition of the respiration, respectively of the GHE and AVEC soils. As the effects of the natural product were statistically small, this bioindicator indicates that the methanolic fraction of the *Polymnia sonchifolia* extract, which has fungicide properties, has no environmental effects.

## 1. Introduction

One of the most important functions of soil microorganisms is the turnover of organic matter that happens mainly by the degradation of plant and animal debris and is reflected in the soil fertility and environmental quality [1–3]. The soil microorganisms mineralize the natural and the added substances that reach this ecosystem, releasing the nutrients required for plant growth, as well as CO_2_ as a byproduct from their respiration [4]. On the other hand, any contaminant or xenobiotic that reaches the soil may interfere with these natural reactions of the biogeochemical processes and their consequences may be not good from the environmental point of view. The N fertilization [5], the soil moisture [6,7], soil temperature [8], and also the presence of pesticide residues in the agricultural environment may disturb the natural degradation processes [9]. Some substrates, including pesticides, may inhibit, but also stimulate the microbial activity [10]. Thus, disturbances of the microbial activity may influence the important biogeochemical processes of the entire soil environment.

Soil microorganisms, although very small, are very active and numerous. Their activities are recognized as important also for the degradation of contaminants, so that tests on biodegradability of pesticides and its influence on the soil microbial activity are required for registration and commercial license of pesticide molecules [1,3,11–14]. The soil microbial activities can be evaluated by the microbial respiration which can be measured by the O_2_ consumption, but the CO_2_ production is considered more sensitive, mainly when ^14^C-compounds are used, because of the sensitivity and accuracy of the detection technique [1,9]. Soil microbial respiration is considered one of the most suitable biosensors because it reflects the cycling of organic matter by the soil microorganisms [8], and is used to measure the changes in the heterotrophic microbial activity caused by pesticide residues. The disturbances are evaluated mainly by the substrate-induced respiration by addition of glucose (SIR) [9,15], and the effects on the CO_2_ production have been used as bioindicator of the pesticide effects on soil microbial activity [16]. The soil respiration is considered so sensitive that a test was developed and adopted by many countries to detect long-term adverse effects of chemicals on the aerobic process of carbon transformation in surface soils [12]. The measurement of the produced CO_2_ is even more accurate when the ^14^C-glucose and biometer flasks are used [17,18].

Chlorothalonil (CAS No. 1897-45-6) as a broad-spectrum organochlorine fungicide is used to control many phytopathogenic fungi that threaten agricultural crops. It is considered moderately or very toxic, moderately persistent in the soil, where the increased moisture or temperature also increase its degradation [19], but some of the commercial formulations of chlorothalonil are classified as very toxic and very dangerous to the environment [20]. It is aerially applied, but some residues may reach and persist in the soil [21], where according the soil type, they may persist for more than 200 days after its application [21,22]. Although the fungicide may be degraded in soil [23], its main metabolite is 30 times more toxic and was more persistent than the parental molecule [24]. These characteristics reinforce the need of search of a natural alternative for persistent synthetic compounds like chlorothalonil.

Some plants have medicinal and antimicrobial properties and their use in agriculture is being studied as an environmentally safer alternative. The aerial parts of *Polymnia sonchifolia*, popularly known as “yacón”, have shown antifungal activity, and pesticides are not needed in its cultivation [25]. The extracts of its leaves inhibited the growth and production of aflatoxin by *Aspergillus flavus* [25,26], and the methanolic fraction showed the best inhibition of fungal growth [27]. Thus, the extract of its leaves has the potential to be a safer alternative to synthetic fungicides.

In order to evaluate the effect of chemicals on the soil environment there are many approaches, including the detection of the adverse effects, the ability of the soil microorganisms to continue metabolizing the organic materials, as well as the effects on the natural processes of carbon transformation by measurements of the CO_2_ released by the biomineralization of organic molecules [9,12,13,28]. The ^14^CO_2_ production from ^14^C-glucose was here used to compare the effect of the natural and synthetic fungicides on the respiration of two different soils.

## 2. Experimental Setup

Analytical-standard chlorothalonil (99.3% of purity) from Riedel-de-Haën (Germany) was used in acetone solution, based in the recommended agricultural application doses. According to the Laboratory of Pharmacology and Chemistry of Natural Products, which extracted the leaves of *Polymnia sonchifolia*, the leaves were dried, crushed and macerated in ethanol during seven days. The solvent was then evaporated till dryness and the resulting residue was submitted to chromatography on a silica gel 60 column which was eluted with hexane, chloroform, ethyl acetate and methanol. The solvents were fully evaporated to give the respective fractions. Solutions of the methanol fraction were freshly prepared in Tween 80 (0.7% in water), just before the application to the soil.

The substrate-induced respiration was performed in 50 g (dry weight) soil samples placed in biometer flasks [17]. The soil samples (Table 1) and the analysis were provided by the Department of Soil Science (School of Agronomy of the São Paulo State University—USP). The soil microbial activity was previously re-activated by re-moistening to 60% of the maximum water holding capacity (MWHC) less one 1.0 mL, one week before the treatments with the active ingredients. Each of two groups (3 × 50 g) of each soil type of soil was treated with an acetone solution of chlorothalonil at 11 μg·g^−1^ soil, or 11 μL acetone·g^−1^ soil, as control soils. The other two groups were treated either with 300 μg·g^−1^ soil of the methanolic fraction of the *Polymnia sonchifolia* extract, or 21.5 μL aqueous solution of Tween 80 (0.7%) g^−1^ soil, as used to dissolve the plant extract. The volumes of acetone and Tween used in the control samples were the same as used with the fungicide treatments.

All the soil samples received 1.0 mL of an aqueous solution of D-[U-^14^C]-glucose (specific activity: 55 mCi·mmol^−1^; Amersham International, UK) and glucose prepared in ultra-pure water (Milli Q). The treatment corresponded to 4.0 mg glucose and 5.16 Bq ^14^C-glucose g^−1^ soil (dry weight), which completed the water volume for 60% WMHC [29]. The side arms of the biometer flasks were filled with 10 mL NaOH (0.1 M), which was changed by newly prepared every two hours during the first 10 hours after the treatments; 24 h after, as well as 3, 5, 7, 14 and 28 days after the treatments. The systems were kept at 25 °C and 12 h light during all the study.

The ^14^C-CO_2_ was analyzed by liquid scintillation counting (LSC) of (at least 5) 2.0 mL sub-samples of the NaOH solution of each replicate. All the values were calculated as percentage of the ^14^C-glucose applied. Results were analyzed by the matched pairs t-test (n ≤ 30 and p ≤ 1%) and the significance of the differences (p = 0.001) was calculated two-to-two with the respective control.

## 3. Experimental Results and Discussion

**Figure 1.**
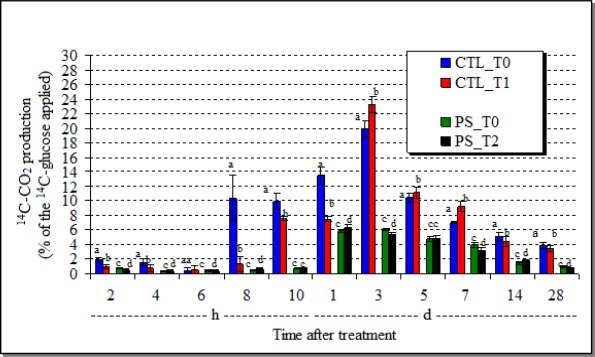
Substrate (^14^C-glucose)-induced respiration of the soil GHE treated either with chlorothalonil (CTL_T1) or the *P. sonchifolia* methanol extract (PS_T2) and their respective controls (CTL_T0; PS_T0), during 28-day incubation. (h = hours; d = days; mean ± SD; different letters near the bars indicate statistical difference in relation to the control).

Although there are pesticides that do not affect soil respiration [30], the GHE soil treated with chlorothalonil presented a statistically smaller production of ^14^C-CO_2_ (p = 0.001) than its untreated control from 8 h to one day after the fungicide treatment (Figure 1). But, afterwards up till 3 days, the presence of chlorothalonil stimulated (p = 0.001) the ^14^C-CO_2_ production, indicating the recovering of the microbial activity, the stimulus by the fungicide and, thus, the enhanced biomineralization of the ^14^C-glucose.

When the GHE was treated with the *P. sonchifolia* methanol fraction, although the ^14^C-CO_2_ production presented statistically different results from the control (p = 0.001), the values were small but indicated a slightly stimulation by the plant extract.

**Figure 2.**
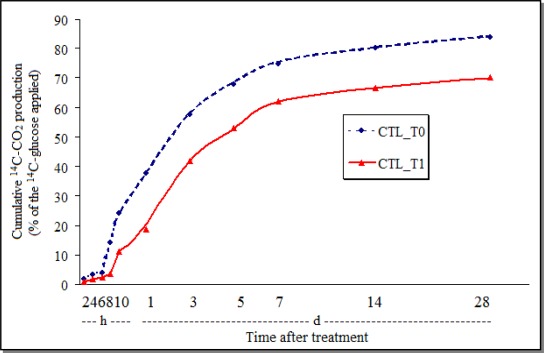
Cumulative production of ^14^C-CO_2_ from ^14^C-glucose by the soil GHE treated with chlorothalonil (CTL_T0 = control; CTL_T1 = chlorothalonil; h = hours; d = days).

As shown with other pesticides [31], chlorothalonil inhibited the GHE-soil respiration and the effect persisted during the 28 days of observation (Figure 2), indicating the interference of the fungicide on the soil-oxidative processes.

**Figure 3.**
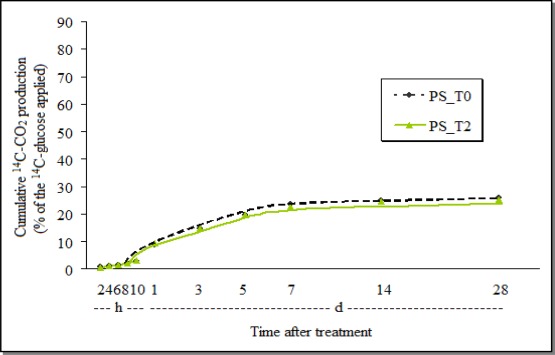
Cumulative production of ^14^C-CO_2_ from ^14^C-glucose by the soil GHE treated with the *P. sonchifolia* methanol fraction (PS_T0 = control; PS_T2 = *P. sonchifolia*; h = hours; d = days) extract.

On the other hand, the cumulative ^14^CO_2_ production after treatment with *P. sonchifolia* extract was very near that of the untreated-control, and just a 2.2% inhibition of ^14^C-glucose biomineralization was observed at the end of the 28 days (Figure 3).

The total biomineralization was much smaller in the control soil samples treated with Tween (around 25%—Figure 3), than in the control treated with acetone (more than 80%—Figure 2). This indicates that the effect of the vehicle of the active ingredient, or that the formulation may also influence the soil biological processes.

**Figure 4.**
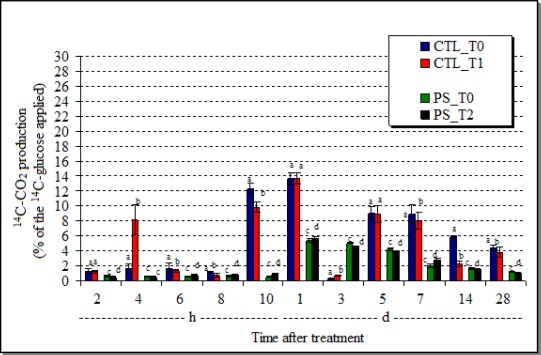
Substrate (^14^C-glucose)-induced respiration of the soil AVEC treated either with chlorothalonil (CTL_T1) or the *P. sonchifolia* methanol fraction (PS_T2) and their respective controls (CTL_T0; PS_T0), during 28-day incubation. (h = hours; d = days; mean ± SD; different letters near the bars indicate statistical difference in relation to the control).

The AVEC-soil respiration was persistently and significantly inhibited (p = 0.001) by chlorothalonil just 7 days after the treatment. The stimulation on the ^14^C-CO_2_ production observed only at 4 h after the treatment (Figure 4) was probably due to intrinsic variations of the microbial activity caused by the homogenization processes of these treated soil samples, but it did not persist.

Although the differences between the values of the ^14^C-CO_2_ production were not very different between the AVEC-soil samples treated and untreated with the *P. sonchifolia* methanol fraction (2 h—Figure 4), the plant extract firstly inhibited (p = 0.001), but thereafter stimulated the ^14^C-glucose respiration until one day after the treatment. Thereafter, the presence of the plant extract inhibited the soil biomineralization of ^14^C-glucose.

**Figure 5.**
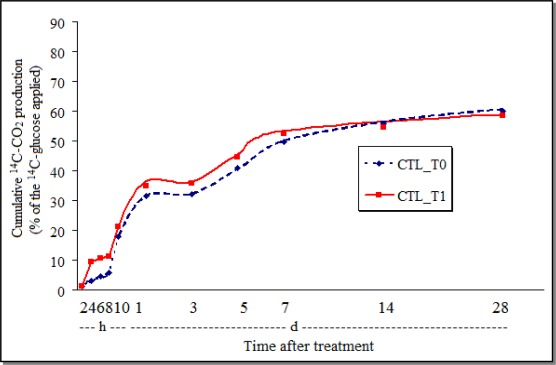
Cumulative production of ^14^C-CO_2_ from ^14^C-glucose by the soil AVEC treated with chlorothalonil (CTL_T0 = control; CTL_T1 = chlorothalonil; h = hours; d = days).

**Figure 6.**
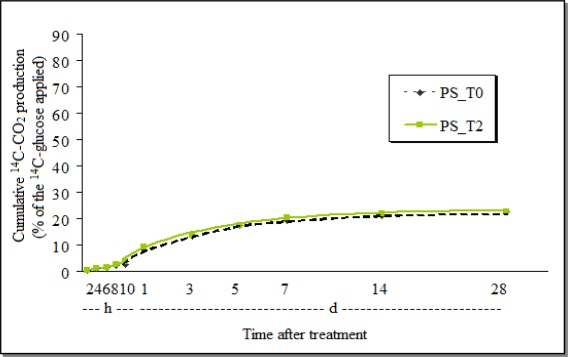
Cumulative production of ^14^C-CO_2_ from ^14^C-glucose by the soil AVEC treated with the *P. sonchifolia* methanol extract (PS_T0 = control; PS_T2 = *P. sonchifolia*; h = hours; d = days) extract.

The cumulative production of ^14^C-CO_2_ after the treatment of the AVEC-soil with chlorothalonil (CTL_T1—Figure 5) demonstrated that initially the fungicide stimulated the respiration, but from around 12 days till the end of the study it was 2.6% inhibited. On the other hand, at the end of the study with the *P. sonchifolia* methanol fraction, the cumulative amount of ^14^C-CO_2_ in AVEC was 1.8% bigger in the soil samples with the plant extract (Figure 6) than in the control. The transient effect of different pesticide and other substances applications on soil microbial respiration has been also detected by others [32,33].

As occurred in the GHE soil, the ^14^C-CO_2_ production was mostly smaller both in the AVEC-soil samples treated with *P. sonchifolia* methanol fraction or its control (around 23%—Figure 6), than the samples treated with chlorothalonil or its control (around 58%—Figure 5). Again, this may indicate that, irrespective of the soil type, just the Tween here used to dissolve the active ingredient caused some inhibition in the soil respiration.

The soil parameters also influenced because, as reported by other authors [33], the ^14^C-glucose biomineralization to ^14^C-CO_2_ increased slowly in both soils during the first hours then increased rapidly, which characterizes the microbial activity. But, the maximum was reached depending mainly on the soil type, *i.e.*, after around 5 days in the GHE soil and around 3 days in the AVEC. The ^14^C-CO_2_ production from the GHE reached more than 65% and from the AVEC it was less than 60%, even with the treatment with chlorothalonil. Although not as large, the biomineralization was also different in the samples treated with the plant extract or with Tween alone, being the highest ^14^C-CO_2_ cumulative production in the GHE—about 25%, and about 22% in the AVEC.

## 4. Conclusions

The methanolic fraction of *Polymnia sonchifolia* extract did not cause great effects in the soils respiration, and can be a good alternative to synthetic fungicides if the plant extract also demonstrates control of phytopathogenic fungi. At the same time this fraction of the plant extract caused approximately the same ^14^C-glucose biomineralization than the vehicle utilized here to dissolve it (Tween), both being less severe than the synthetic pesticide chlorothalonil on the biomineralization processes, which indicates the need to find a safer formulation. On the other hand, the soil microbiota was able to produce great amounts of ^14^C-CO_2_, even in the presence of the synthetic chlorothalonil.

## Figures and Tables

**Table 1.** Main physical and chemical characteristics of soils.

**Soil**	**pH (H_2_O)**	OMC*	**Clay**	**Silt**	**Sand**
			**g·kg^−1^**		
Eutrophic Humic Gley-GHE	5.5	13	154	142	704
Typic Eutroferric Chernosol-AVEC	6.2	42	480	210	310

* organic matter content.
